# Metabolic pairing of aerobic and anaerobic production in a one-pot batch cultivation

**DOI:** 10.1186/s13068-018-1186-9

**Published:** 2018-07-03

**Authors:** Milla Salmela, Tapio Lehtinen, Elena Efimova, Suvi Santala, Rahul Mangayil

**Affiliations:** Laboratory of Chemistry and Bioengineering, Tampere University of Technology, Korkeakoulunkatu 8, Tampere, Finland

**Keywords:** Synthetic microbial consortia, Metabolic pairing, Integrated metabolism, Hydrogen production, Wax esters

## Abstract

**Background:**

The versatility of microbial metabolic pathways enables their utilization in vast number of applications. However, the electron and carbon recovery rates, essentially constrained by limitations of cell energetics, are often too low in terms of process feasibility. Cocultivation of divergent microbial species in a single process broadens the metabolic landscape, and thus, the possibilities for more complete carbon and energy utilization.

**Results:**

In this study, we integrated the metabolisms of two bacteria, an obligate anaerobe *Clostridium butyricum* and an obligate aerobe *Acinetobacter baylyi* ADP1. In the process, a glucose-negative mutant of *A. baylyi* ADP1 first deoxidized the culture allowing *C. butyricum* to grow and produce hydrogen from glucose. In the next phase, ADP1 produced long chain alkyl esters (wax esters) utilizing the by-products of *C. butyricum*, namely acetate and butyrate. The coculture produced 24.5 ± 0.8 mmol/l hydrogen (1.7 ± 0.1 mol/mol glucose) and 28 mg/l wax esters (10.8 mg/g glucose).

**Conclusions:**

The cocultivation of strictly anaerobic and aerobic bacteria allowed the production of both hydrogen gas and long-chain alkyl esters in a simple one-pot batch process. The study demonstrates the potential of ‘metabolic pairing’ using designed microbial consortia for more optimal electron and carbon recovery.

**Electronic supplementary material:**

The online version of this article (10.1186/s13068-018-1186-9) contains supplementary material, which is available to authorized users.

## Background

Almost all known biochemical reactions can be found in microbes, making them the most metabolically diverse class of organisms in the world. The natural versatility of microbes, and on the other hand, the development of cell engineering technologies have enabled their utilization in a great number of applications ranging from bioremediation of xenobiotics to the production of complex drug precursors [1–3]. Despite the advancements in cell-based processes enabled by metabolic engineering and synthetic biology, the laws of thermodynamics and the nature of organisms limit the number and efficiency of different metabolic pathways in a single organism. For example, the anaerobic fermentation of sugars to short-chain alcohols or organic acids is energetically favourable and redox balanced for cells, which makes the process scale-up rather straight forward. As a trade-off, however, in these catabolic “downhill” pathways the substrate is converted to a product with lower carbon content, causing substantial carbon loss in the form of unwanted side products. In contrast, the synthesis of many industrially relevant products with long carbon chain, such as alkanes, fatty acids, and alkyl esters, is energetically expensive for cells, and thus harnessing such thermodynamically “uphill” synthesis pathways for a profitable process is challenging. Recent advances in exploiting microbial electrosynthesis (MES) hold great potential for the energy recovery by external electron transfer, but further efforts are required to overcome the challenges related to the limitations of metabolic capabilities and systematic strain engineering for greater efficiency [4, 5].

A potential strategy for combining a broader range of metabolic attributes is to utilize two or more different species or strains in a single process. In recent years, growing attention has been given to rationally designed microbial consortia addressing the issues related to the constraints of a single cell, such as limited substrate range or biosynthetic efficiency. For example, Hanly and Henson modelled substrate conversion by simultaneous utilization of hexoses and pentoses in a cocultivation by a respiratory deficient *Saccharomyces cerevisiae* and *Scheffersomyces stipitis* [6]. In another example, Minty et al. demonstrated the production of isobutanol directly from lignocellulose hydrolysate by a coculture of a fungi *Trichoderma reesei* and a bacterium *Escherichia coli*. In the culture, *T. reesei* secreted cellulases to create solubilized sugars, which were utilized by *E. coli* and further converted to isobutanol [7]. Park et al. also used cellulase-excreting fungi in a coculture. In their study, *Acremonium cellulolyticus* was used in a one-pot approach together with *Saccharomyces cerevisiae* to produce ethanol [8]. Similarly, Wang et al. used a bacterium *Clostridium celevecrescens* together with *Clostridium acetobutylicum* ATCC824 to produce butanol [9]. Zhang et al. on the other hand, reported a coculture system, which involved a partial distribution of the metabolic pathway for *cis*–*cis*-muconic acid production in two engineered *E. coli* strains [10]. As a result of efficient intermediate redirection and alleviated burden to cells, the production of *cis*–*cis*-muconic acid was significantly improved.

Other typical issues related to single-cell cultures include excessive byproduct formation and poor tolerance against certain feedstock components and/or the (by)product itself. Organic acids, such as acetate, impose a great challenge in bioprocesses both in terms of toxicity to cells and as a drain for carbon and energy [11]. We have previously demonstrated the benefits of acetate redirection to biomass and product in a consortium of *E. coli* and *Acinetobacter baylyi* ADP1 [12]. In the culture, engineered *A. baylyi* ADP1 consumed the acetate produced by *E. coli*, which improved the biomass production and recombinant protein expression compared to single-cell cultures. More recently, we have successfully combined MES with long-chain ester synthesis by sequentially culturing an acetogenic strain *Sporomusa ovata* and *A. baylyi* ADP1 [13]. In the separated two-stage process, the acetogenic strain utilized carbon dioxide and electricity to produce acetate, which was subsequently fed to ADP1 to produce long-chain alkyl esters. In a somewhat reverse culture set-up, we established a coculture of *A. baylyi* and *Clostridium butyricum* for the production of hydrogen (H_2_) gas from simulated and rice straw hydrolysates [14]. The glucose-negative *A. baylyi* selectively removed the *C. butyricum* growth inhibitors, namely acetate, formate, 4-hydroxybenzoate, and oxygen from culture, allowing the subsequent growth and H_2_ production by *C. butyricum* from hydrolysate sugars.

In the present study, we demonstrate the successful integration of anaerobic fermentation (representing the ‘downhill’ pathway) to aerobic synthesis (the ‘uphill’ pathway). In the process of two metabolically diverse bacterial species, aerobic and anaerobic phases alternate allowing the conversion of carbon and electrons to biomass and products in a non-optimized simple batch culture. As an ultimate example of a ‘carbon wasting’ downhill process, glucose is utilized as a substrate for biohydrogen production by the obligate anaerobe *C. butyricum*. The fermentation byproducts, namely acetate and butyrate, together with naturally occurring oxygen, are converted to highly energy and carbon-rich alkyl esters with an average of 34-carbon chains by the strict aerobe *A. baylyi* ADP1. This study demonstrates the potential of combining distinctive bacterial metabolisms for optimal electron and carbon recovery.

## Results

**Fig. 1:**
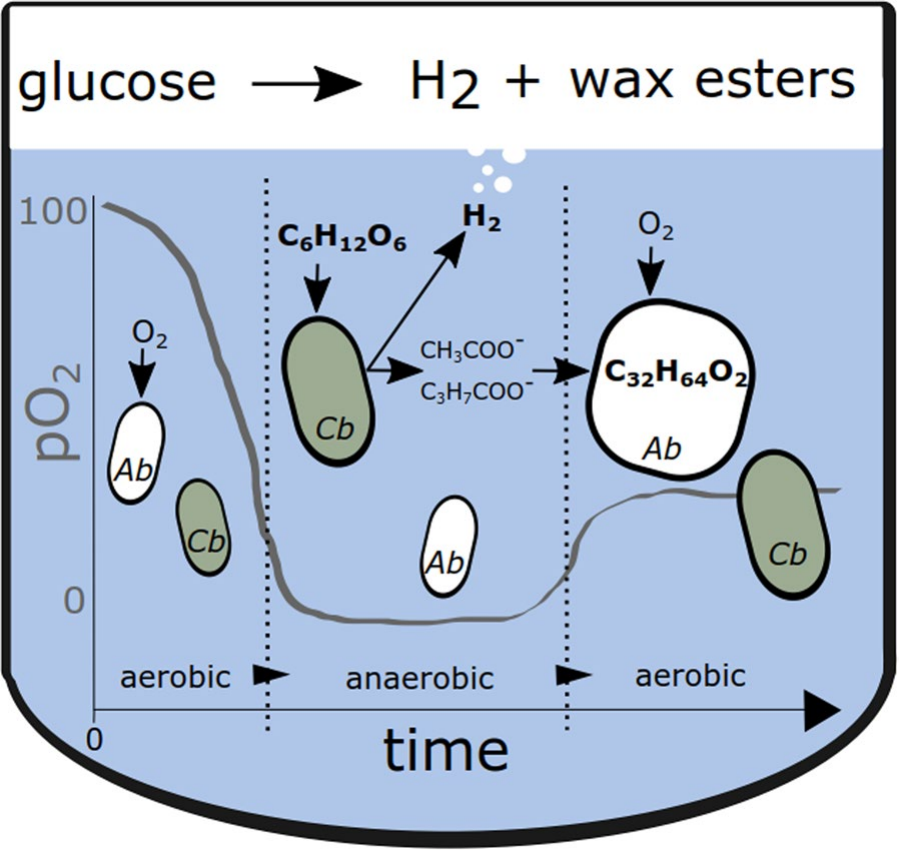
Schematic representation of the one-pot batch cultivation of *C. butyricum* and ADP1-g strain. In the first stage, the metabolic activity of ADP1-g deoxidizes the culture, allowing H_2_ production from glucose by *C. butyricum*. Thereafter, oxygen is released to the culture, enabling wax ester production from the residual carbon, namely acetate and butyrate, by ADP1-g

To allow efficient distribution and comprehensive consumption of carbon in a cocultivation of *C. butyricum* and *A. baylyi*, a previously engineered knock-out strain of *A. baylyi* deficient in glucose consumption was deployed [14]. The strain *A. baylyi* ADP1 Δgcd, designated here as ADP1-g, lacks the gene for glucose dehydrogenase, which is responsible for the first reaction in the modified Entner–Doudoroff pathway of *A. baylyi* sugar catabolism [15]. We hypothesized that in the cocultivation *C. butyricum* consumes the glucose and produces H_2_, followed by the production of long chain alkyl esters by ADP1-g from the liquid end-metabolites (acetate and butyrate) of *C. butyricum* (Fig. 1). In the first stage of the process, the growth medium is deoxidized by the metabolic activity of ADP1-g strain, allowing the growth and H_2_ production of the obligate aerobe *C. butyricum*. After *C. butyricum* growth, oxygen is allowed to enter the system, enabling ADP1-g growth and wax ester production from the residual carbon, namely acetate and butyrate.

### Suitability of ADP1-g for the alternating bioprocess conditions

**Fig. 2:**
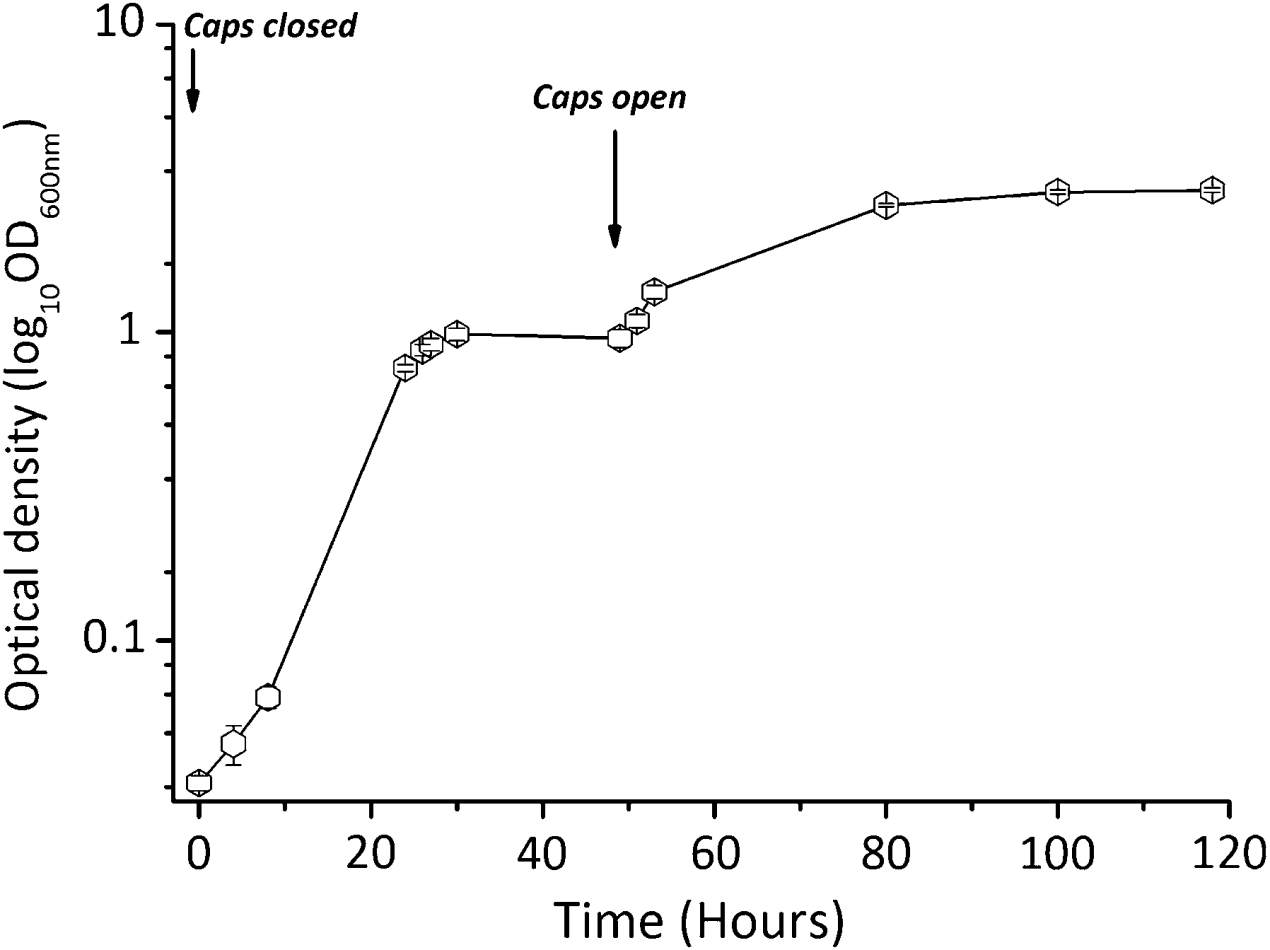
ADP1-g growth during alternating oxygen availability. The ability of the strain ADP1-g to withstand anoxic environment was conducted in 10 ml of initial aerobic MA/9 medium. Upon inoculation, the cultivation tubes were closed until growth cessation (30 h) and opened at 50 h time-point to resume the growth. The data points and error bars represent mean value and standard deviation from triplicate experimental repeats, respectively. In some cases, the error bars are smaller than the symbol

We investigated the capability of the obligate aerobe ADP1-g to survive and subsequently grow in cultivation with varying oxygen availability. In this experiment, ADP1-g was cultivated with limited oxygen supply in sealed vessels. The cells grew for 30 h, after which the growth ceased due to oxygen depletion (Fig. 2). These anoxic growth conditions were maintained for another 24 h and then oxygen was released to the vessels. Shortly after, an exponential increase in cell density was observed demonstrating that ADP1-g maintains cell viability after exposure to oxygen-deprived conditions for 24 h.

**Fig. 3:**
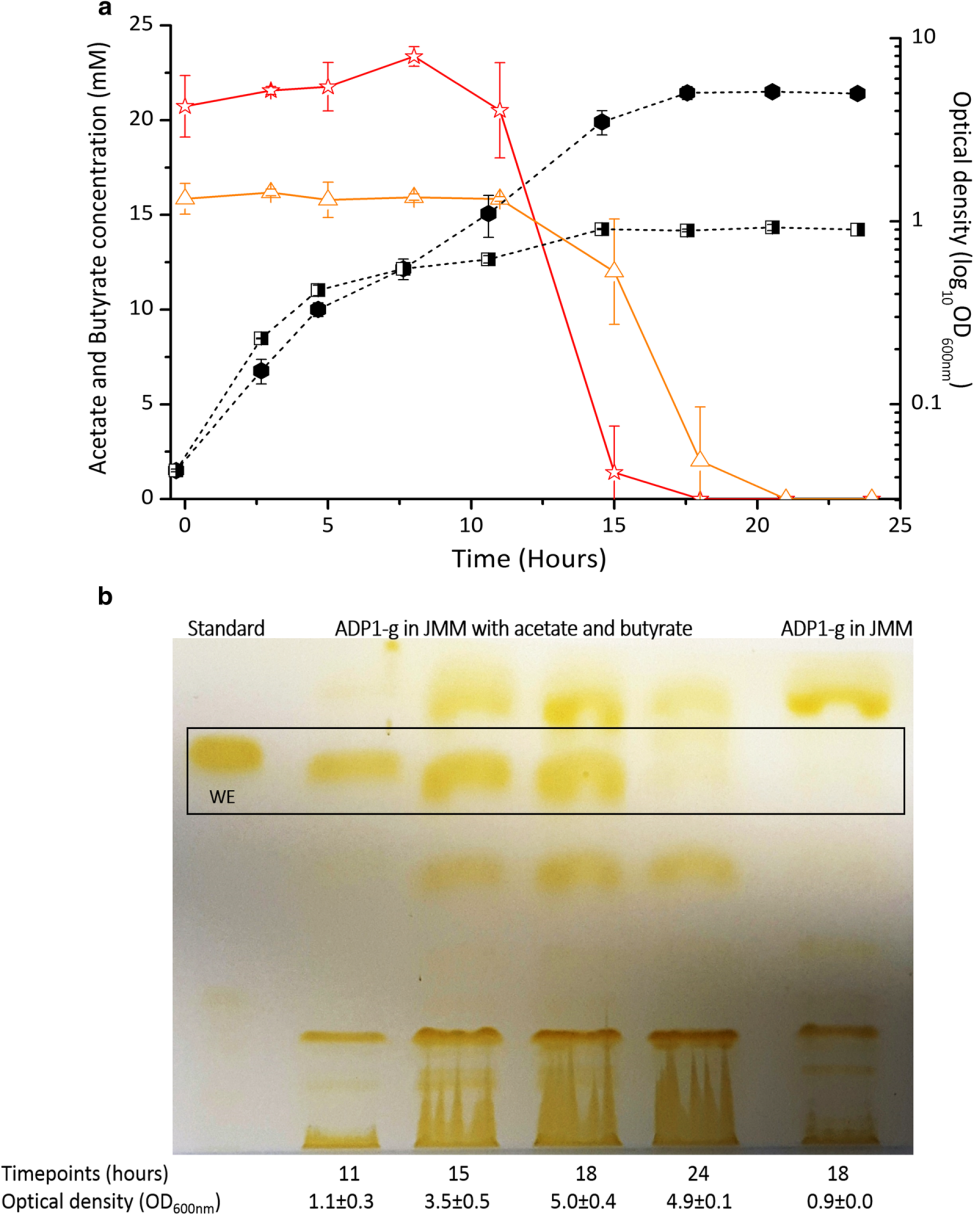
Growth, substrate utilization and wax ester production of aerobic ADP1-g cultivations with 20 mM acetate and 15 mM butyrate in 50 ml JM medium. **a** The growth (closed circles), determined as optical density at 600 nm wavelength, and organic acid utilization (acetate, open stars; butyrate, open triangles) by ADP1-g is presented. The data points are averaged values and error bars indicate standard deviation from triplicate experimental repeats. In some cases, the error bars are smaller than the symbol. **b** Thin layer chromatography analysis of wax ester synthesized by ADP1-g from acetate and butyrate. The time point samples analyzed with TLC correspond to the same presented in (**a**). In above figures a and b, ADP1-g cells in JM medium (including 0.3 g/l of yeast extract and 25 µg/l of chloramphenicol diluted in ethanol) represents the growth (black and white square) and wax ester profiles, respectively, of ADP1-g cells when cultivated without acetate and butyrate supplementation

Another growth test was conducted with ADP1-g to investigate the utilization of acetate and butyrate, which are potential end-products of *C. butyricum* in the cocultivation system. The strain consumed both carbon sources (initial 15 mM butyrate and 25 mM acetate) within 21 h (Fig. 3a) reaching an optical density of 5.1 ± 0.3. In addition, wax esters were produced and accumulated up to 18 h of cultivation (Fig. 3b). Thereafter, the amount of WEs decreased due to the carbon depletion, which is a typical phenomenon in carbon-limiting conditions [16]. A control cultivation in JM media reached an optical density of 0.9 ± 0.0 (Fig. 3a) with insignificant wax ester production (Fig. 3b) compared to the cultivation supplemented with acetate and butyrate indicating that these acids are used for wax ester production by ADP1-g.

### Cocultivation of ADP1-g and *C. butyricum* in batch

In the next stage, ADP1-g and *C. butyricum* were cocultivated in a 300 ml batch culture. In this one-pot approach, aerobic and anaerobic phases alternate allowing both bacteria to grow. Only one cycle of aerobic–anaerobic–aerobic phase was employed due to the near complete utilization of substrate during the first anaerobic phase. Although being characterized as an obligate anaerobe, *C. butyricum* is known to tolerate oxygen [17]. However, it ceases to grow until exposed back to anaerobic conditions. In addition, as a spore forming bacteria, it survives even prolonged exposure to high oxygen pressure. Based on this knowledge and the results regarding ADP1-g viability after exposure to anaerobic conditions, in our experiments both strains were inoculated simultaneously to the incubation vessel. Anoxic conditions were produced and maintained by the metabolic activity of the aerobic ADP1-g. During this process, *C. butyricum* remains inactive in relation to growth due to the aerobic environment, whereas after deoxygenation the metabolic activity of *C. butyricum* is initiated. The metabolite formation by *C. butyricum*, as well as the growth and end-products of both *C. butyricum* (produced during the anaerobic phase) and ADP1-g (produced in the aerobic phase), were determined. Twenty millimolar of glucose was used as carbon source for *C. Butyricum* growth based on preliminary tests conducted with different substrate concentrations (Additional file 1: Table S1). Ten millimolar acetate supplementation was chosen for initial growth and deoxygenation by ADP1-g according to ADP1-g acetate utilization trend obtained from preliminary studies conducted with ADP1-g alone (Additional file 2: Table S2).

**Fig. 4:**
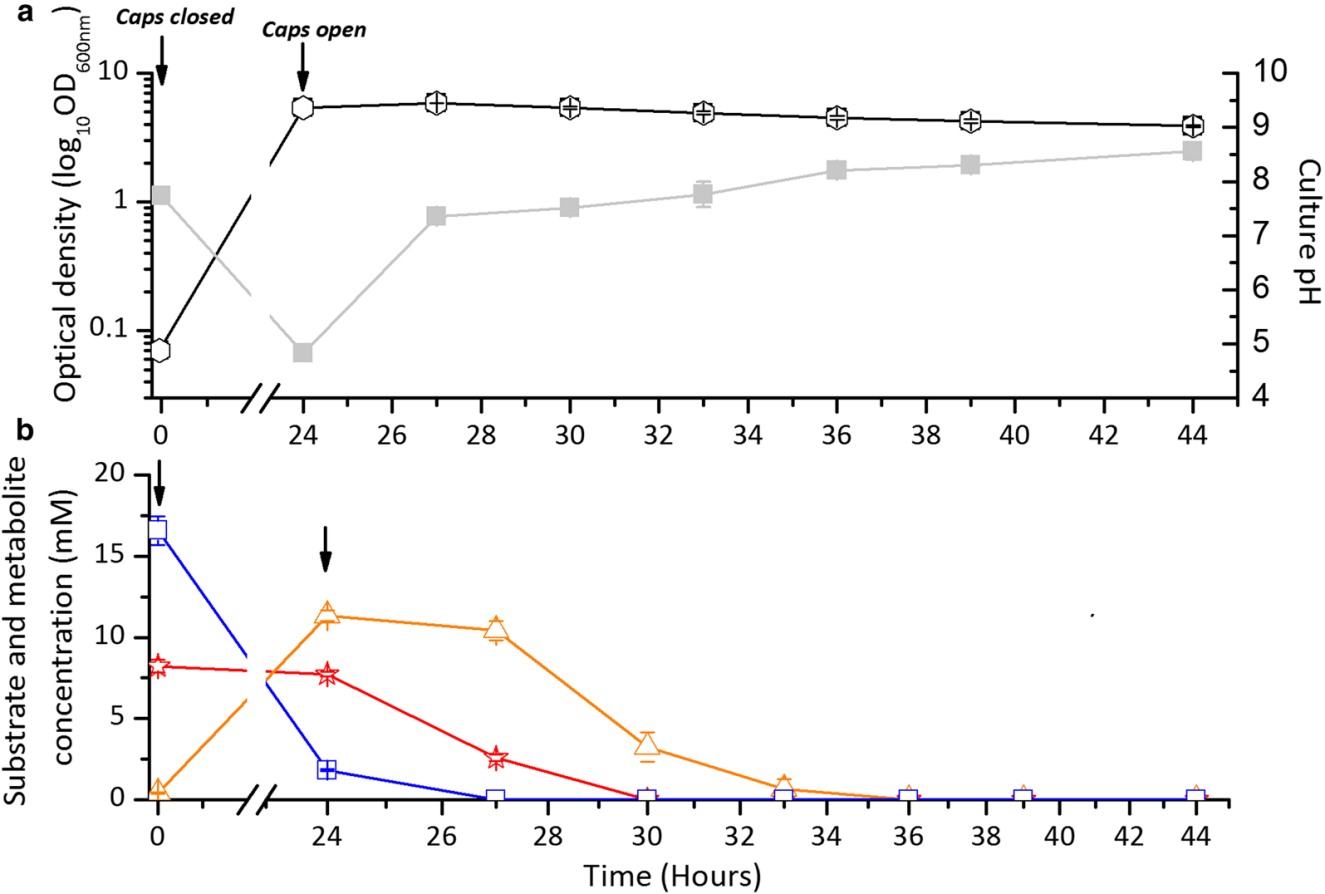
The growth, pH, and substrate concentrations of ADP1-g—*C. butyricum* cocultivations in 300 ml JM medium. Arrows indicate the time when the growth vessel caps were closed (for anaerobic phase) and opened (for second aerobic phase) during the experiment. **a** OD_600nm_ (open circle) and pH (closed square) trends of one-pot batch ADP1-g—*C. butyricum* cocultivations. **b** Glucose utilization (*C. butyricum,* open square) and acetate–butyrate metabolism (acetate, open star; butyrate, open triangle) data from cocultivation experiment. The data points are averaged from triplicate experimental repeats. In some cases, the symbols overlap and the error bars (standard deviation) are smaller than the symbol

During the anaerobic phase, *C. butyricum* produced 1.6 ± 0.1 mol H_2_/mol glucose with a cumulative H_2_ production of 131.5 ± 5.2 ml. A drop in pH associated with *C. butyricum* growth occurred (pH 5) and most of the supplemented glucose was consumed (Fig. 4a, b). Additionally, 11.4 ± 0.3 mM of butyrate and 7.7 ± 0.1 mM of acetate were produced from the *C. butyricum* fermentation (Fig. 4b).

**Fig. 5:**
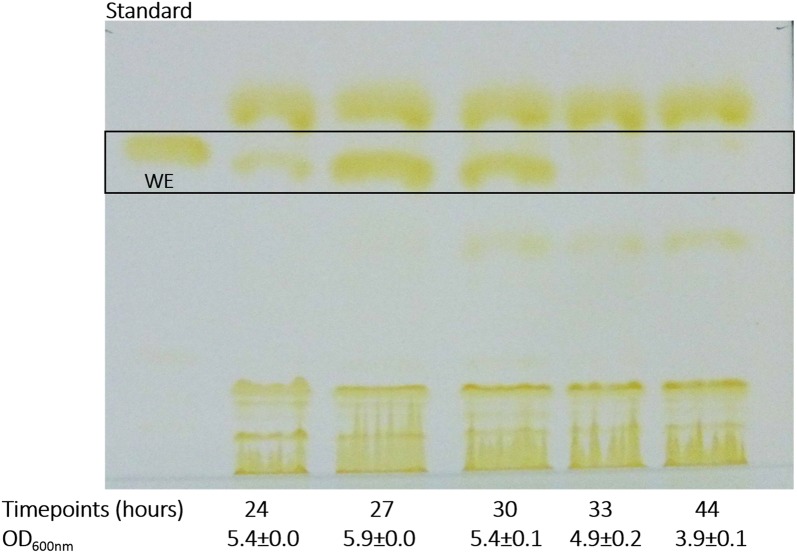
TLC analyses demonstrating the wax ester production from ADP1-g—*C. butyricum* coculture. The wax esters were synthesized by ADP1-g strain utilizing the acetate and butyrate generated during *C. butyricum* fermentation. The samples are from the same timepoints shown in Fig. 4

After the *C. butyricum* fermentation, oxygen was released to the system enabling ADP1-g growth. The pH was also adjusted (pH 7) to ensure optimal growth for ADP1-g. Wax esters, the end products of ADP1, accumulated during 24–30 h of cultivations (Fig. 5). During this time, acetate was completely consumed along with most of the butyrate (Fig. 4b). The WEs were solely produced by ADP1-g, as *C. butyricum* does not produce WEs (Additional file 3: Figure S1).

**Fig. 6:**
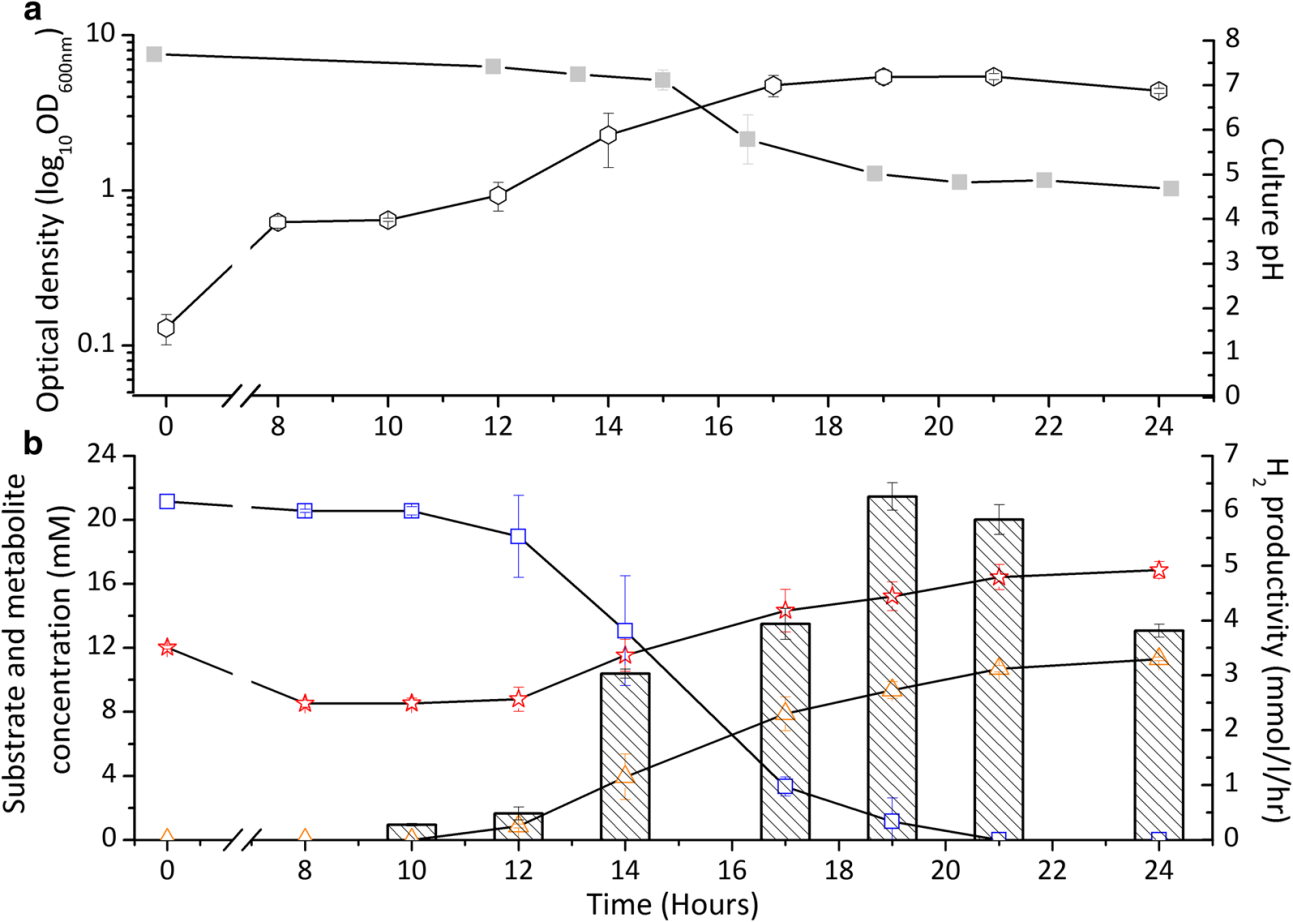
The growth, pH, substrate and metabolite concentrations and hydrogen production from the aerobic–anaerobic phases of the ADP1-g—*C. butyricum* cocultivations in 300 ml JM medium. Initial aerobic conditions turned to anaerobic via ADP1-g deoxygenation within 12 h of cultivation initiating glucose consumption and hydrogen production by *C. butyricum*. **a** OD_600nm_ (open circle) and pH (closed square) trends of one-pot batch ADP1-g—*C. butyricum* cocultivations. **b** Glucose utilization (*C. butyricum,* open square), acetate–butyrate metabolism (acetate, open star; butyrate, open triangle) and hydrogen productivity mmol/l/h per culture volume (in bars) from cocultivation experiment. The data points are averaged from triplicate experimental repeats. In some cases, the symbols overlap and the error bars (standard deviation) are smaller than the symbol

To determine the hydrogen production during the coculture of ADP1-g and *C. butyricum* as a function of time, additional 300 ml batch cocultures supplemented with 20 mM of glucose and 10 mM of acetate were carried out. Samples collected during the first aerobic and the following anaerobic phase were analyzed for substrate consumption, as well as end product and metabolite formation. Within 12 h, the conditions were made anaerobic as is indicated by the ceased growth of ADP1-g and the initiation of glucose consumption, hydrogen production and metabolite formation by *C. butyricum* (Fig. 6a, b). During the anaerobic phase, glucose was completely utilized and a significant increase in cell density was observed. From the consumed glucose, 4.6% of the electrons were recovered in H_2_ during 21 h cultivation time. The maximum hydrogen productivity of 6.3 mmol/l/h was reached after 19 h of cultivation and 8.3 mM of acetate and 11.3 mM of butyrate were produced by *C. butyricum* in the anaerobic phase (Fig. 6b). As expected *C. butyricum* did not grow in aerobic JM medium (Additional file 4: Figure S2). This demonstrates the efficiency of such cocultivation system to deoxygenize and facilitate fermentative hydrogen production by *C. butyricum* in a medium devoid of reducing agents.

### Cocultivation of ADP1-g and *C. butyricum* in a 1-l bioreactor

**Fig. 7:**
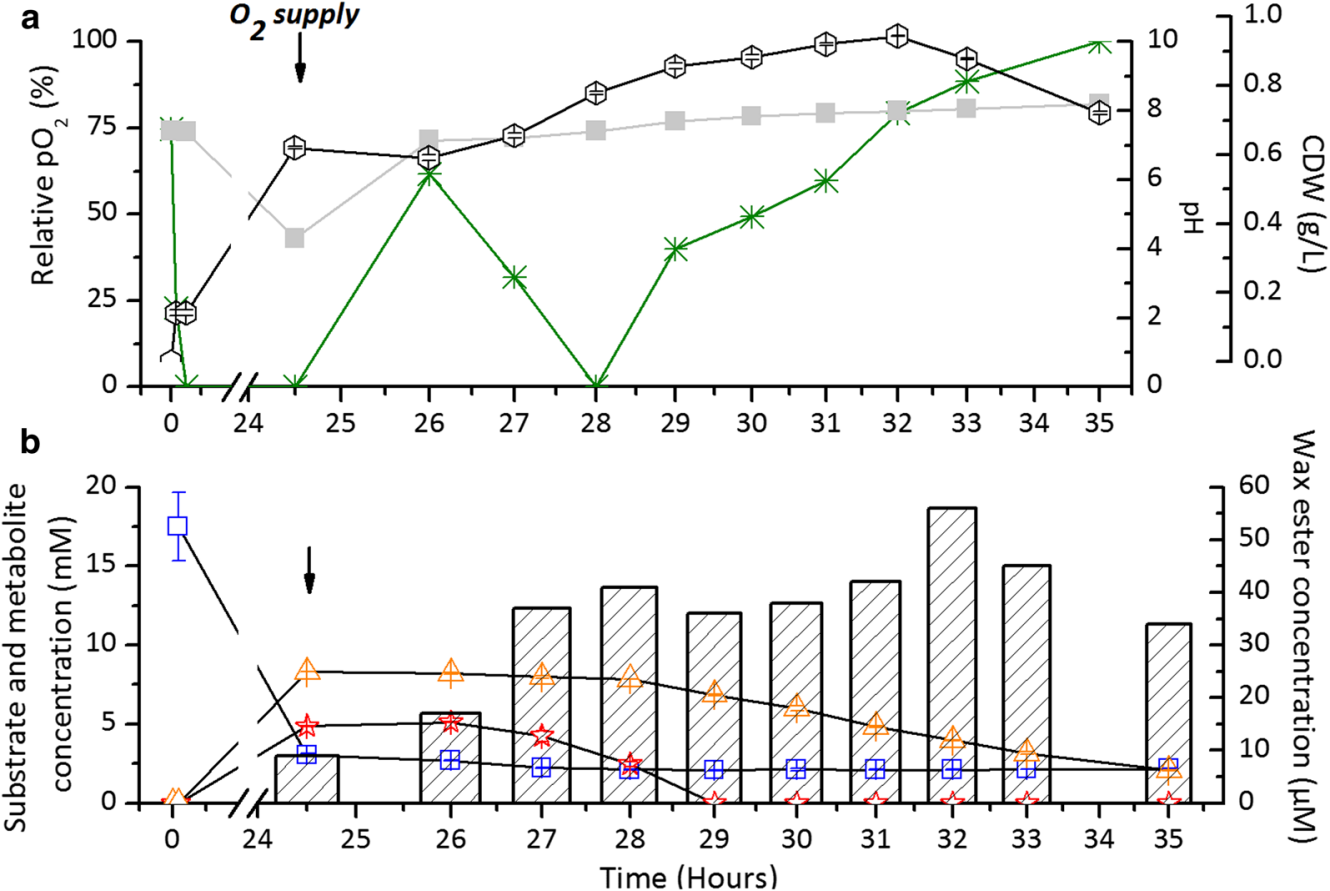
One-pot batch cultivation of ADP1-g and *C. butyricum* in 1-l bioreactor. The cocultivation was carried out in 800 ml of initially aerobic JM medium supplemented with 20 mM glucose. The changes in (**a**) pH (closed square), pO_2_ (asterisk) and biomass formation (in cell dry weight, CDW; open circle), and (**b**) glucose (open square), acetate (open star), butyrate (open triangle) and wax ester concentrations (in bars) are presented. *C. butyricum* consumed glucose producing acetate and butyrate during the first 24 h (anaerobic phase), whereas ADP1-g utilized the fermentation end-products during the following 10 h (aerobic phase). Arrows indicate the timepoint at which the oxygen supply was initiated and the culture pH adjusted to 7.3. The data for CDW, glucose, acetate and butyrate are mean values from triplicate technical repeats. The standard deviations (error bars) of each data are plotted and in some cases the symbols overlap the error bars

For a more detailed analysis of the coculture, a cultivation experiment was conducted in a 1 l bioreactor. Similarly to the previous batch cultivation, ADP1-g and *C. butyricum* were simultaneously inoculated in the culture and the cultivation vessel was sealed. Oxygen partial pressure, pH, substrate/metabolite concentrations, and cell growth were monitored during the cultivation. Hydrogen was measured from the total gas collected during the anaerobic phase of the cultivation and wax ester production was quantitatively measured by NMR from the collected samples. The culture was deoxygenated by ADP1-g within 5 min after the inoculation (Fig. 6a) and anoxic conditions were maintained for the next 24 h. During this anaerobic phase, *C. butyricum* consumed most of the supplemented glucose (83%) producing 4.8 mM of acetate, 8.3 mM of butyrate and 24.5 ± 0.8 mmol/l H_2_. The products generated in this process are shown in Table 1 with respect to concentrations, productivities, titers and yields. The metabolic activity of *C. butyricum* ceased when oxygen was released to the system causing a small amount of glucose to remain in the medium. The carbon and electrons from the utilized glucose (14.5 mM) were recovered in the metabolites and end products of *C. butyricum* and the recovery percentages were calculated as 96.9 and 106.5%, respectively (Additional file 5: Table S3). Within 3 h of the latter aerobic phase, an increase in cell dry weight was observed as ADP1-g strain started to grow. During this aerobic stage, the strain recovered the carbon from *C. butyricum* metabolites for cell growth and storage compounds, namely wax esters (Fig. 7a and b, Table 1). Acetate was consumed rapidly in 4.5 h, whereas butyrate was consumed more gradually (Fig. 6b). The highest wax ester yield (30 mg/g biomass) was achieved after 8 h of aerobic cultivation (32 h of overall cultivation) with 100% of acetate and 52% of butyrate consumed. The total yields of products recovered from the batch cocultivation were 1.7 mol H_2_/mol glucose_consumed_ and 10.8 mg WE/g glucose_consumed_, respectively.

**Table 1:** Products generated from glucose in one-pot batch cultivation by *C. butyricum* and ADP1-g

	Substrate utilized and products generated	Yield
Substrate		
Glucose	14.5 ± 2.1^a^	
Products		
Biomass	0.94^b^	
Lipids	9.7^c^	
H_2_	24.5 ± 0.8^d^	1.7 ± 0.1^e^
WE	28^d^	10.8^e^
		30^f^
		31^g^

^a^Fermented glucose (Initial glucose–residual glucose), mmol/l. The data represents mean value from duplicate technical repeats ± standard deviation

^b^Biomass, g/l

^c^Total lipid content in biomass, %

^d^H_2_ and WE concentration in mmol/l and mg/l, respectively. The H_2_ production data represents averaged value from triplicate GC measurements ± standard deviation

^e^H_2_ and WE yield from glucose, mol H_2_/mol glucose_consumed_ and mg WE/g glucose_consumed_. H_2_ yield represents the mean yield calculated from triplicate GC measurements and averaged glucose concentration ± standard deviation

^f^WE yield per gram biomass, mg WE/g biomass

^g^WE content in total lipids, %

## Discussion

Efficient recovery of carbon and electrons in the final product is crucial in biological production processes. For cells, however, the optimal balance between carbon utilization and redox state often requires the by-production of unwanted metabolites. Moreover, the product range may be constrained depending on the metabolic capabilities of the cell. To address these issues, we designed and constructed a ‘metabolic pair’ of anaerobic and aerobic metabolisms enabling both fermentative hydrogen production and aerobic lipid synthesis with enhanced carbon and energy recovery in a simple one-pot batch cultivation. In the coculture set-up, a strict anaerobe *C. butyricum* was deployed for the production of H_2_ gas using glucose as the source of carbon and energy, whereas the strictly aerobic ADP1-g was utilized for maintaining oxygen-free conditions during the anaerobic fermentation, followed by the recovery of any residual carbon in the culture for the aerobic production of wax esters. In the batch process carried out in “one-pot”, alternating aerobic and anaerobic phase allowed consecutive growth of the bacteria.

For the coculture system to be functional, the aerobic strain is required to both survive and sustain anaerobic conditions during the H_2_ production phase. Previously, ADP1-g has been shown in a proof of concept experiment to be able to deoxygenize growth media for *C. butyricum* [14], albeit the viability of the strain to survive anoxic conditions was not investigated. Thus, in our first experiment, the ability of ADP1-g to restore growth after anoxic phase was studied. According to the growth data, we found out that the strain was able to continue growth after being incubated in oxygen-limited conditions for 24 h. In the subsequent experiments, we also confirmed that the strain is able to uphold sufficient anaerobic conditions during the oxygen-sensitive *C. butyricum* growth and H_2_ production phase. During the aerobic and anaerobic phases, only one of the strains was growing at a time, as *C. butyricum* does not grow in the presence of oxygen. ADP1-g, on the other hand, requires oxygen for growth. Examples of other approaches for employing oxygen-consuming bacteria as deoxygenizing agents have been previously reported. For example, cocultures for maintaining anoxic conditions for *C. butyricum* growth and H_2_ production (in a culture initially made anaerobic) have been made with *Enterobacter* [18]. Other cocultivation approaches have included systems to remove oxygen for *C. butylicum* or *C. acetobutylicum* growth with the help of *Bacillus subtilis* to increase butanol yields [19, 20]. However, as the aerobic species do not contribute to product formation in these processes, the substrate utilized for their metabolism is “wasted” and lowers the yield of the overall process. In contrast, in our system, ADP1-g not only consumes the oxygen, but also scavenges and upgrades the otherwise wasted organic acids produced by *C. butyricum*. Notably, ADP1-g does not consume glucose, and thereby does not impair the efficiency of H_2_ production from glucose.

The incapability of ADP1-g to utilize glucose has been previously demonstrated [14]. Wax ester production by *A. baylyi* ADP1 using acetate as a carbon source has been also previously reported [21] whereas WE production from butyrate has not been recorded to our knowledge. In our study, ADP1-g was shown to grow and produce alkyl esters (WEs) from both acetate and butyrate, which makes it ideal for upgrading the metabolites of *C. butyricum*. It is also notable that the strain was able to continue growth and establish wax ester production directly in the medium ‘used’ and occupied by *C. butyricum*.

*Clostridium butyricum* ferments glucose and other carbohydrates readily to H_2_ and volatile fatty acids (VFA), such as acetate and butyrate [22, 23]. Thermodynamic and metabolic restrictions in biological systems divert the substrate also towards biomass and other metabolites [24]. Thus, the theoretical yields of the NADH-dependent H_2_ production by *Clostridium* spp. when the sole liquid fermentation metabolites are acetate and butyrate vary between 2 and 4 mol-H_2_/mol-glucose depending on the ratio of produced metabolites butyrate and acetate. As the latter is associated with higher H_2_ yields, and *Butyricum* typically favors butyrate production, the actual yields obtained are closer to the lower range of the theoretical values. In our experiments, the cocultivation yielded 1.7 ± 0.1 mol H_2_/mol glucose_consumed_ with an acetate to butyrate ratio of 0.6:1. As comparison, experiments with pure cultures of *C. butyricum* in optimized production conditions have yielded results such as 2.29 mol H_2_/mol glucose [23] and 2.21 mol H_2_/mol glucose [22] whereas coculture designs have yielded values such as 1.65 mol H_2_/mol glucose [25]. The metabolites (acetate and butyrate) produced in our experiments account up to 49.5% of the carbon and 59.1% of the electrons from the initial glucose supplementation, which are an untapped resource when using monocultures. In this experiment, *A. baylyi* consumed 4.8 mM acetate and 4.3 mM butyrate for the production of 56 µM WE. An average WE molecule in *A. baylyi* has 34 carbons [26]. Thus, the c/c yield from acetate and butyrate to WE was 7%. This is a bit higher than the yield obtained with acetate as the sole carbon source (4.4%) [13]. In our study, the organic acids were scavenged in the second aerobic stage of the one-pot system providing additional value for the process in the form of 10.8 mg WE/g glucose_consumed_.

The WE yield of the overall process (10.8 mg/g glucose_consumed_) is competitive when compared to other microbial WE production systems. As an example, the yeast *S. cerevisiae* has been recently reported to produce very long chain WEs from glucose with the yield of 0.75 mg/g glucose [27]. Another example is the production of branched chain WEs with engineered *E. coli* from glucose (with additional supplementation with propionate, n-octanol and oleic acid), with the yield of 2.5 mg/g glucose [28]. In our earlier study, we produced WEs with engineered *A. baylyi* from glucose with the yield of 40 mg/g glucose [26]. In the present study, the *A. baylyi* strain was not engineered with respect to WE production, and the culture conditions were not optimized for WE production, underlining the good performance of the coculture with respect to other published production systems.

In a system where both strains are grown separately, the culture conditions can be optimized according to each microorganism’s preferences for single end product formation. In contrast, a ‘one-pot’ approach limits optimization parameters depending on the requirements of both of the microorganisms used. However, such cocultures provide other benefits whilst maintaining the bioprocess competitive. Sustainable and cheap substrates, such as lignocellulosic biomass, restrict culture medium optimization by default due to complex structure, inhibitory compounds and pH. In one pot systems, such as used in this study, the culture media can be detoxified by ADP1-g for C*. Butyricum* growth and product formation. In the present study, the detoxification is limited to oxygen removal, though ADP1-g could be used to remove inhibitory compounds from the media as was demonstrated in [14]. In a two phase system, the benefits of the first phase of the “one-pot” system (detoxification) would be lost.

Co-culture systems allow the design of production systems with metabolic capabilities exceeding those of any individual organism. Zhou et al. [29] produced oxygenated taxanes by dividing the complex pathway between *E. coli* and *S. cerevisiae*. *E. coli* produced taxadiene, which was oxygenated by *S. cerevisiae*, resulting in the production of complex oxygenated taxane molecules that have not been produced by any single microbe. In another example, Liu et al. [30] used a consortium consisting of three different microbes for the division of labor to enhance the electricity production from glucose. In the consortium, *E. coli* first converted glucose to lactate, which was oxidized by *Shewanella oneidensis* to acetate. The acetate was further utilized by *E. coli*. *B. subtilis*, in turn, produced riboflavin, which is required as an electron shuttle. These studies exemplify how a single biosynthetic pathway can be divided between different organisms to improve the production. In the present study, we combine two very different metabolisms (aerobic and anaerobic, catabolic and anabolic) that complement each other for the efficient recovery of electrons (H_2_ and WE) and carbon (WE).

The relevance of the current platform could be further improved by engineering the production efficiency and/or product range. *A. baylyi* ADP1 can be readily engineered for improved production of native or non-native products [21, 30–33], whereas limited number of studies describing successful engineering of *C. butyricum* has been reported. Thus, one of the advantages of metabolically diverse cocultures is the modularity and flexibility of the system; substrate and product range can be individually engineered and tuned for applicable strain(s). Such division of engineering load lowers the metabolic burden in one strain, and on the other hand, reduces the challenges faced with strains for which engineering tools and methods are not readily available.

## Conclusions

Cocultivation of organisms with divergent metabolic characteristics can greatly broaden the substrate and product range and facilitates more comprehensive utilization of carbon and energy. We successfully integrated bacterial anaerobic and aerobic metabolisms in a synthetic consortium, and by exploiting the advantageous properties of both metabolisms, demonstrated the production of hydrogen gas and long-chain alkyl esters in a single process. The study demonstrates the power of metabolic pairing in resolving metabolic and thermodynamic limitations of single organisms, as well as in developing novel metabolic combinations for more efficient carbon and energy recovery.

## Methods

### Strains and cultivation medium

*Acinetobacter baylyi* ADP1 (DSM 24193) devoid of glucose dehydrogenase gene ACIAD2983, (referred here as ADP1-g) and *Clostridium butyricum*, isolated from a hydrogen-producing bioreactor [34], were used in the study. Unless otherwise indicated, ADP1-g cells cultivated in low-salt Lysogeny broth (LB) (tryptone, 10 g/l; yeast extract, 5 g/l; sodium chloride, 1 g/l) at 30 °C and 300 rpm, were used as pre-inoculums. The growth kinetics of ADP1-g in a three stage bioprocess was studied in in MA/9 medium (g/l; 5.518 Na2HPO_4_·2H_2_O, 3.402 KH_2_PO_4_, 1 NH_4_Cl, 0.008 nitrilotriacetic acid, 0.487 FeCl_3_, 0.25 MgSO_4_·7H_2_O, 0.02 CaCl_2_·2H_2_O, 0.2% casein amino acids and 2 ml/l of SL7 trace element solution). ADP1-g acetate and butyrate utilization experiments were conducted in modified minimal medium referred as JM media [14] ((g/l): 1.5 K_2_HPO_4_, 2.0 (NH_4_)_2_SO_4_, 0.2 MgSO_4_*7H_2_O, 0.015 CaCl_2_*2H_2_O, 0.005 FeSO_4_*7H_2_O, 0.3 yeast extract). *C. butyricum* precultivations were conducted in anaerobic Reinforced Clostridial Medium (RCM, Sigma Aldrich, US) at 37 °C and 150 rpm. The co-cultivation experiments, using ADP1-g and C*. butyricum*, were conducted in JM medium and the cultures were grown at 30 °C and 300 rpm. Chloramphenicol (25 µg/ml, diluted in ethanol) was used as an antibiotic in experiments conducted by ADP1-g alone.

### Experimental procedure

The growth test to investigate ADP1-g viability in the three stage aerobic–anaerobic–aerobic bioprocess was conducted in tubes containing 10 ml of sterile aerobic MA/9 medium. Upon inoculating (initial optical density 0.02), the cultivation tubes were closed with sterile septum rubber stoppers, tightened with aluminum crimps and incubated at 30 °C and 300 rpm. A blank cultivation (devoid of inoculant) was included as control to determine ‘false positive’ results from contamination and as analytical control. The experiment was conducted in triplicates and the cell growth data measured at specific intervals using a spectrophotometer (Ultrospec 500 pro, Amersham Biosciences, UK) for 120 h, were averaged.

Batch experiments to investigate ADP1-g capacity to utilize organic acids from *C. butyricum* fermentation was performed in 120 ml serum bottles with a working volume of 50 ml sterile JM medium. Acetate and butyrate were supplemented at concentrations 20 mM and 15 mM, respectively. The inoculated cells (initial cell density 0.02) were cultured aerobically at 30 °C and 300 rpm. The cells were also inoculated to a substrate blank, i.e. cultivation medium devoid of acetate and butyrate. Samples to analyze cell growth, organic acid utilization, lipid analysis and medium pH were collected in 3-h interval for 24 h.

The co-cultivations of ADP1-g and *C. butyricum* were conducted in triplicate 500 ml batch bottles containing 300 ml of sterile aerobic JM medium. To initiate ADP1-g and *C. butyricum* growth, the medium was supplemented with 10 mM acetate and 20 mM glucose, respectively. Two millilitre of precultivated ADP1-g with an optical density at wavelength 600 nm (OD_600nm_) of 3.2 was inoculated alone and co-inoculated with 2 ml of *C. butyricum* precultivation (OD_600nm_ 3.1). ADP1-g cultivated in similar medium and a blank medium were used as the controls in this experiment. Following inoculation, the batch bottles were capped (rubber stoppers), tightened (aluminum crimps). Initial samples to determine cell growth, substrate utilization, liquid metabolites and lipids were collected and the cultures were incubated at 30 °C and 200 rpm. The gaseous end products were analyzed from the bottle headspace after a 24 h cultivation period and the rubber stoppers were removed. Opening the bottle caps ensured the end of anaerobic phase and culture samples for analysis were collected. The medium pH, after the anaerobic phase, from the coculture, ADP1-g alone and blank cultivations were measured aseptically and adjusted to pH 7.4 (initial pH) with 5 M sterile sodium hydroxide solution. The cultures were divided to sterile 50 ml serum bottles and incubated under aerobic conditions at 30 °C and 200 rpm. The experiment was conducted in triplicates and samples were collected every 3 h during 20-h cultivation for analysis.

Additional cocultivation experiments to determine the hydrogen production as a function of time were conducted in 500 ml bottles as above, except samples were collected for cell growth, gaseous product analyses, substrate consumption and metabolite formation periodically during the anaerobic phase and the experiment was ended before the second aerobic phase. *C. butyricum* alone and ADP1-g alone cultivations were used as controls. Cell growth of the ADP1 alone cultivation was monitored to determine the starting point for sampling (OD, pH, GC and HPLC analysis) from the cocultivation.

Bioreactor experiment was conducted in a sterile 1-l vessel (Sartorius Biostat B plus Twin System, Germany) with an online pH and pO_2_ monitoring system. To a medium volume of 795 ml, 50 ml of ADP1-g precultivated in JM medium supplemented with 25 mM acetate (OD_600nm_ 3.9) and 5 ml of *C. butyricum* precultivated in RCM (OD_600nm_ 2.3) were inoculated, totaling the cultivation volume to 850 ml. To prevent gas leakage, the fermentor outlets were closed and a gas collection bag (Supelco, USA) was connected to the exhaust. After initial sample collection, the cultures were grown at 30 °C and 350 rpm for 24 h. At the end of anaerobic phase, the collection bag was removed, samples were collected for analysis and the medium pH was adjusted at 7.3 with sterile 5 M NaOH solution. The aerobic phase was initiated with air supply. The medium pH and partial O_2_ (pO_2_) profiles were obtained from the bioreactor. From the bioreactor, 45 ml of culture was removed every hour from which duplicate technical repeats to monitor cell growth and organic acid utilization were collected and the remaining culture (~ 40 ml) was used for quantitative lipid analysis.

### Analytical techniques

For qualitative lipid analysis, 4 ml of culture samples were analyzed using thin layer chromatography as described in [16]. Briefly, the samples were centrifuged and the cell pellet was suspended in methanol and chloroform. After centrifugation, the lower chloroform phase was applied on the TLC plates (10 × 10 cm Silica Gel 60 F_254_ HPTLC glass plates with 2.5 × 10 cm concentrating zone, Merck, USA) and visualized with iodine. For dry cell weight (DCW) calculations in bioreactor experiment, 40 ml of original cultures were pelleted in pre-weighed tubes at 30,000*g* (4 °C), freeze-dried and weighed. The tube weights were subtracted from the freeze-dried cell weights to determine the DCW (g/l). The lipids extracted from the freeze-dried cells were quantitatively analyzed using NMR as described in [16]. Briefly, the WE content of the cells was quantified from extracted total lipids by ^1^H NMR measurements (Varian Mercury spectrometer, 300 MHz). The spectra were taken from samples dissolved in chloroform using trifluorotoluene as an internal standard. The obtained data were processed using ACD NMR processor program and interpreted accordingly. NMR allows quantitative detection of α-alkoxy methylene protons of alcohols specific for wax esters.

Glucose, acetate and butyrate concentrations were analyzed, in triplicates, using high-performance liquid chromatography (HPLC) (LC-20AD, Shimadzu, Japan) equipped with Shodex SUGAR (SH1011) column (300 × 8 mm), refractive index detector (RID, RID-10A) and 0.01 N H_2_SO_4_ as mobile phase. One millilitre of culture samples were centrifuged at 15,000*g* for 7 min (4 °C), filtered through 0.2 µm polycarbonate filter (Chromafil^®^ PET-45/25, Macherey–Nagel, Germany) and diluted tenfold with ultrapure water in 1 ml HPLC vials. The injection volume, column temperature and mobile phase flowrate was set to 100 µl, 40 °C and 0.6 ml/min, respectively. Identification and quantification of carbon substrate and liquid fermentation metabolites were based on chromatography using external standards.

A gas chromatograph (GC-2014, Shimadzu GC) fitted with a thermal conductivity detector, PORAPAK column (2 m × 2 mm) and N2 as carrier gas was used to analyze the gaseous end products as described in [35]. In brief, the GC maintained with column, oven and detector temperatures of 80, 80 and 110 °C, respectively, 200 µl of sample from the collection bag was injected to the sampling port. The overpressure, i.e. gas volume exceeding the headspace volume of the vessel, was analyzed using water displacement method. The H_2_ and CO_2_ concentrations (mM) were calculated by dividing the cumulative gas (H_2_/CO_2_) volume (ml, obtained from the GC data, injection volume and gas overpressure values) with the product of ideal gas law constant (for room temperature) and cultivation volume (l). Hydrogen yields (mol H_2_/mol glucose_consumed_) were calculated by dividing the H_2_ concentration (mM) with the utilized sugar (mM). In H_2_ productivity studies, the cumulative hydrogen production (ml) profile was calculated as described previously [36]. Hydrogen productivities (mmol/l/h) were calculated by converting the cumulative hydrogen volume to concentration and dividing it with sampling time interval. For batch experiments, each experimental repeats were measured twice and in bioreactor experiment, triplicate technical repeats were included.

Carbon balances of *C. butyricum* gaseous and liquid metabolites and utilized substrate were calculated by multiplying the concentrations with the respective number of carbon in the molecular formula. The CO_2_ in the liquid phase was ignored during the carbon balance calculations. Electron balances were calculated by multiplying the carbon in utilized glucose and each metabolite with the corresponding degree of reduction (mol electrons per C-mol). For carbon and electron mass calculations, the chemical formula of *C. butyricum* biomass was assumed to be CH_1.624_O_0.456_N_0.216_P_0.033_S_0.0047_ [37]. The biomass concentrations (mM) after anaerobic cultivation was calculated by dividing the DCW with the total mass from the formula (g/mol). Both carbon and electron recoveries were calculated by determining the percentage of sum total of carbon and electron mass of liquid and gaseous metabolites divided with the respective masses calculated for the utilized sugar.

## Additional files

**Additional file 1: Table S1.** Substrate utilization and H_2_ production profiles from *C. butyricum* and ADP1-g—*C. butyricum* coculture grown in 10 ml aerobic JM medium supplemented with glucose and 10 mM acetate. Data represents the average from triplicate experimental repeats ± standard deviation.

**Additional file 2: Table S2.** Growth, pH and carbon utilization trend of ADP1-g cultivated in JM medium supplemented with glucose and acetate in aerobic conditions.

**Additional file 3: Figure S1.** Thin layer chromatography analysis of wax ester production from pure cultures of ADP1-g, *C. butyricum* and their cocultures when grown in aerobic and anaerobic JM medium containing varying glucose amounts.

**Additional file 4: Figure S2.** Growth trend of ADP1-g alone and *C. butyricum* alone control cultivations in 300 ml batch cultivations. ADP1-g growth on supplemented acetate (open circles) in initially aerobic conditions and *C. butyricum* growth (closed triangles) without ADP1-g deoxygenation.

**Additional file 5: Table S3.** The carbon and electron masses (mmol) and distribution (%) of *C. butyricum* biomass, gaseous and liquid metabolites generated from glucose in the anaerobic phase in 1-L bioreactor are presented.
