# Supplementary material for: Metabolic pairing of aerobic and anaerobic production in a one-pot batch cultivation
Source: Biotechnol Biofuels. 2018 Jul 3;11:187. doi: 10.1186/s13068-018-1186-9 (PMC6029424; doi:10.1186/s13068-018-1186-9)
Supplement: Supplementary file 1 — Additional file 1: Table S1. Substrate utilization and H2 production profiles from C. butyricum and ADP1-g—C. butyricum coculture grown in 10 ml aerobic JM medium supplemented with glucose and 10 mM acetate. Data represents the average from triplicate experimental repeats ± standard deviation. [file 13068_2018_1186_MOESM1_ESM.docx]

| **Cultures** | **Glucose concentration (mM)** | **Glucose utilization (%)** | **H_2_ production (mM)** | **H_2_ yield (mol / mol glucose_consumed_)** |
| --- | --- | --- | --- | --- |
| *C. butyricum in anaerobic JM medium* | | | | |
|  | 0 | - | - | - |
|  | 10 | 100 | 21.4±0.6 | 2.1±0.1 |
|  | 20 | 70.0±1.0 | 36.5±0.1 | 1.8±0.0 |
|  | 50 | 38.0±2.0 | 37.7±4.0 | 0.8±0.1 |
| *ADP1-g – C. butyricum coculture in aerobic JM medium* | | | | |
|  | 0 | - | - | - |
|  | 10 | 100 | 13.0±0.2 | 1.3±0.0 |
|  | 20 | 100 | 47.0±1.4 | 2.4±0.1 |
|  | 50 | 84.0±2.0 | 59.5±3.2 | 1.2±0.1 |
