# Supplementary material for: Metabolic pairing of aerobic and anaerobic production in a one-pot batch cultivation
Source: Biotechnol Biofuels. 2018 Jul 3;11:187. doi: 10.1186/s13068-018-1186-9 (PMC6029424; doi:10.1186/s13068-018-1186-9)
Supplement: Supplementary file 2 — Additional file 2: Table S2. Growth, pH and carbon utilization trend of ADP1-g cultivated in JM medium supplemented with glucose and acetate in aerobic conditions. [file 13068_2018_1186_MOESM2_ESM.docx]

| **Cultivation time (hours)** ^a^ | **Growth (OD_600nm_)** ^b^ | **Medium pH** ^b^ | **Glucose concentration (mM)** ^b^ | **Acetate concentration (mM)** ^b^ |
| --- | --- | --- | --- | --- |
| 0 | 0.1±0.0 | 7.8±0.0 | 17.2±0.2 | 8.5±0.1 |
| 24 | 0.7±0.0 | 7.3±0.0 | 17.1±0.4 | BD |
| 27 | 1.1±0.1 | 7.9±0.1 | 17.6±1.4 | BD |
| 44 | 1.0±0.1 | 8.5±0.1 | 17.4±0.3 | BD |

^a^ Sampling time similar as indicated in Figure 4

^b^ Averaged data from triplicate experimental repeats ± standard deviation

BD, below detection limit
