# Supplementary material for: Metabolic pairing of aerobic and anaerobic production in a one-pot batch cultivation
Source: Biotechnol Biofuels. 2018 Jul 3;11:187. doi: 10.1186/s13068-018-1186-9 (PMC6029424; doi:10.1186/s13068-018-1186-9)
Supplement: Supplementary file 5 — Additional file 5: Table S3. The carbon and electron masses (mmol) and distribution (%) of C. butyricum biomass, gaseous and liquid metabolites generated from glucose in the anaerobic phase in 1-L bioreactor are presented. [file 13068_2018_1186_MOESM5_ESM.docx]

| **Species** | **Concentration (mM)** | **Carbon (mM)** | **Carbon distribution (%)** | **Electrons (mM)** | **Electron distribution (%)** |
| --- | --- | --- | --- | --- | --- |
| ***Substrate*** | | | | | |
| Glucose (C_6_H_12_O_6_) ^a^ | 14.46 | 86.76 |  | 347.04 |  |
| ***Products*** | | | | | |
| Acetate (C_2_H_3_O_2_^-^) | 4.86 | 9.73 | 11.6 | 38.92 | 10.5 |
| Butyrate (C_4_H_7_O_2_^-^) | 8.31 | 33.24 | 39.5 | 166.19 | 44.9 |
| Hydrogen (H_2_) | 24.48 | - | - | 48.97 | 13.2 |
| Carbon dioxide (CO_2_)^b^ | 21.2 | 21.2 | 25.2 | - | - |
| Biomass (CH_1.624_O_0.456_N_0.216_P_0.033_S_0.0047_)^c^ | 19.87 | 19.87 | 23.6 | 115.65 | 31.3 |
| Total Carbon mass |  | 84.04 |  |  |  |
| Total Electron mass |  |  |  | 369.72 |  |
| Carbon and electron recovery (%) |  | 96.87 |  | 106.54 |  |

^a^ Fermented glucose = (Initial glucose - residual glucose)

^b^ CO_2_ in the liquid phase was ignored.

^c^ P in biomass formula ignored
